# Anal incontinence, urinary incontinence and sexual problems in primiparous women – a comparison between women with episiotomy only and women with episiotomy and obstetric anal sphincter injury

**DOI:** 10.1186/s12905-014-0157-y

**Published:** 2014-12-16

**Authors:** Mona Stedenfeldt, Jouko Pirhonen, Ellen Blix, Tom Wilsgaard, Barthold Vonen, Pål Øian

**Affiliations:** The National Advisory Unit on Continence and Pelvic Floor Health, University Hospital of North Norway, Tromsø, Norway; Department of Clinical Medicine, Women’s Health and Perinatology Research Group, Faculty of Health Science, University of Tromsø, Tromsø, Norway; Research Group Maternal, Reproductive and Children’s Health, Faculty of Health, Oslo and Akershus University College of Applied Sciences, Oslo, Norway; Department of Community Medicine, Faculty of Health Science, University of Tromsø, Tromsø, Norway; Department of Obstetrics and Gynecology, University Hospital of North Norway, Tromsø, Norway

**Keywords:** Obstetric anal sphincter injuries, Episiotomy characteristics, Anal incontinence, Urinary incontinence, Sexual problems

## Abstract

**Background:**

Obstetric anal sphincter injuries (OASIS) might cause anal incontinence (AI) and sexual dysfunction, and might be associated with urinary incontinence (UI). Episiotomy has been identified both as a risk and a protective factor of OASIS. Lately, episiotomies with specific characteristics have shown to be protective against the risk of OASIS. However, little is known about episiotomy characteristics and pelvic floor dysfunction. This study investigates AI, UI, and sexual problems in primiparous women with episiotomy, comparing women with and without OASIS. Associations between episiotomy characteristics and AI, UI, and sexual problems were assessed.

**Methods:**

This is a matched case–control study investigating 74 women with one vaginal birth, all with an episiotomy. Among these, 37 women sustained OASIS and were compared to 37 women without OASIS. The two groups were matched for vacuum/forceps. AI, UI and sexual problem symptoms were obtained from St. Mark’s scoring-tool and self-administered questionnaires. The episiotomy characteristics were investigated and results assessed for the whole group.

**Results:**

The mean time from birth was 34.5 months (range1.3-78.2) for those with OASIS and 25.9 months (range 7.0-57.4) for those without OASIS, respectively. More women with OASIS reported AI: 14 (38%) vs. 3 (8%) p = 0.05 (OR 4.66, 95% CI 1.34-16.33) as well as more problem with sexual desire p = 0.02 (OR 7.62, 95% CI 1.30-44.64) compared to women without OASIS. We found no association between episiotomy with protective characteristics and dysfunctions.

**Conclusion:**

Women with OASIS had more AI and sexual problems than those without OASIS. Episiotomy characteristics varied greatly between the women. Episiotomy with protective characteristics was not associated with increased dysfunctions. OASIS should be avoided, and correct episiotomy used if indicated.

## Background

Obstetric anal sphincter injury is a serious complication of vaginal delivery that can cause significant morbidity, leading to anal incontinence (AI) in 30 - 50% of the women despite adequate repair [1-4]. These injuries can cause sexual dysfunctions [2], and might be associated with urinary incontinence (UI) [5]. Postpartum AI, UI, and sexual dysfunctions are all distressing health problems and have potentially detrimental effects on the quality of life [6-8].

Episiotomy is one of the most frequently used obstetric procedure as well as one of the most debated. There are great variations in both techniques and indications amongst midwives and obstetricians [9,10] and there are conflicting results regarding the association between episiotomy and OASIS [11-13]. The most common episiotomy techniques described in the literature are midline and mediolateral technique. Midline episiotomy starts at the posterior fourchette followed by a straight downward cut, the mediolateral episiotomy starts at the posterior fourchette and continues with a cut 40-60° from the midline [14]. Lateral episiotomy is rarely described even though studies show that lateral episiotomy is a tradition in some European countries [15-17], and commonly performed unintentionally [9,11,16]. Lateral episiotomy starts to the left or right of the midline, at either 4–5 or 7–8 o’clock and the cut is angled 40–60° from the midline [14].

Research has found that clinicians very often perform mediolateral episiotomy improperly [12,16,18,19]. This is important because a correct mediolateral episiotomy with an angle of 40-60° is shown to have protective properties and decrease the risk of sustaining OASIS compared to episiotomies with narrower angles [12,18]. In a recent case–control study, we sought to assess associations between episiotomy characteristics and OASIS and found that a lateral episiotomy with correct angle, sufficient length and depth reduced the risk of sustaining OASIS compared to mediolateral episiotomy [18].

Episiotomy is suggested to be associated with postpartum dyspareunia and perineal pain [20,21]. Further, mediolateral episiotomy is thought to cause more perineal pain and dyspareunia compared to midline episiotomy [22]. On the other hand, a recent study found no difference in perineal pain between women with midline, mediolateral and lateral episiotomy when the episiotomy technique used was investigated 0–3 days after delivery [16].

The primary aim of this study was to assess if there were differences in prevalence of AI, UI and sexual problems in women with episiotomy and OASIS compared to women with episiotomy only. Secondly, to assess if episiotomy characteristics were associated with AI, UI and sexual problems.

## Methods

This study was the second part of a matched case–control study carried out at the University Hospital of North Norway and Nordland Hospital (permission for both hospitals: Regional Ethics Committee of North Norway (163/2008) [18]. Included in the study were primiparous women with OASIS who had episiotomy and clinically identified tears graded as 3a, 3b, 3c, or 4 [23], and women without OASIS who had episiotomy only. Procedures for diagnosing OASIS are similar at both units; if the midwife or physician responsible for the delivery suspect a sphincter tear, a specialist in obstetrics and gynecology confirms it, and the repair is done by an experienced obstetrician or colorectal surgeon [18].

The women were identified through the hospitals electronic patient journal system Partus® (CSAM Health AS, Lysaker, Norway). Women were matched for ventouse/forceps because of the strong association between OASIS and instrumental delivery. Fifty-three women with OASIS and 75 matched control women were eligible. The women were contacted and asked to participate by letter and phone-call. Five women were excluded because of language or pregnancy and 16 OASIS cases and 33 matched controls, a total of 49 women, declined to participate. Seventy-four eligible women (70% of eligible cases and 50% of eligible controls) were willing to participate in the study [18].

**Figure 1:**
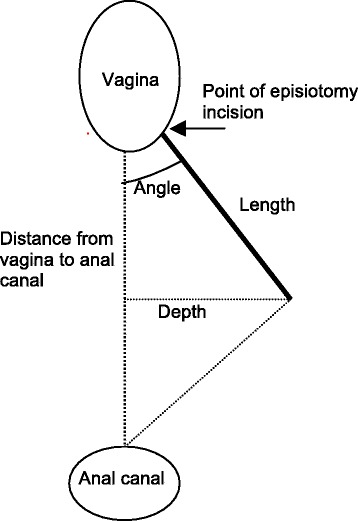
**Adapted from Andrews et al. 2005** [19] **and Stedenfeldt et al. 2012** [18] **and illustrates the episiotomy measurements taken.**

After the women had signed an informed consent form, they were called in for a physical examination [18]. During the consultation, the introitus vagina/perineum was investigated for the episiotomy scar. One of the authors (MS) did the investigation and photography at mean 34.5 and 25.9 months after birth for women with and without OASIS, respectively. The photography was standardized with the women in stirrups and the camera in fixed positions [18]. Based on the photos taken, the episiotomy length, incision point, depth, distance from the anal canal, and angle given between the fixed points of the posterior fourchette, the episiotomy, and the most anterior point of the anal epithelium were measured (Figure 1) [18].

To differentiate the episiotomies based on the incision point they were categorized into hours as in an analog clock display: 6, 6–5, 5, 5–4, 4, 4–3, 3. Incision points equal to 6 and between 6–5 were categorized as medial incision point, and incision point above 5 was categorized as lateral incision point. Further, the episiotomies were categorized into angles ranging as: <15, 16–30, 31–45, 46–60, >60°.

Bowel, urinary, and sexual problems were registered by questionnaires and scoring systems. AI was defined as an involuntary loss of flatus, liquid, or solid. Symptoms were registered with the St. Mark’s scoring system, which is a validated interview tool consisting of seven questions for grading the severity of AI during the final four weeks [24]. The scores range from 0 to 24. In this study, the severity of symptoms was graded according to clinical relevance: 0–3 = “no AI”; 4-8 = “mild/moderate”; ≥9 = “severe”. The scores were also dichotomized so that 0-3 = “no AI” and ≥4 = “AI”. MS carried out all the interviews and scorings. UI was defined as a symptomatic involuntary loss of urine and documented by the validated self-administered questionnaire ICIQ-UI SF, which was developed for assessing the type of UI as well as its prevalence, severity, and impact on quality of life [25,26]. The answers resulted in a sum ranging from 0 to 21. Scores were also dichotomized so that 0 = “no UI” and ≥1 = “UI” [25]. Furthermore, according to clinical relevance, UI was categorized as 1-8 = “mild” and ≥9 = “moderate/severe”.

Sexual problems were evaluated by questions originally used by the Norwegian Institute of Public Health to assess sexual problems in Norway [27]. The women were asked whether they were sexually active, with the response options being “YES” or “NO”. If the answer was “YES”, the degree of sexual problems was measured by the question: “Have you experienced any of the sexual problems listed below during the past 12 months/or since sexual activity was retained after birth”. The following three problems were listed: 1) reduced sexual desire, 2) orgasm problem, 3) experience of genital pain during intercourse. Response categories for each of the items were 0 = “not active due to problems”, 1 = “problem all the time”, 2 = “problem nearly all the time”, 3 = “problem quite often”, 4 = “problem quite rarely”, and 5 = “never problem”.

Data were analyzed using SPSS version 18 (SPSS Inc, IL, USA). Conditional logistic regression models were used to assess differences between women with and without OASIS in the rate of AI, UI, and sexual problems. Spearman’s correlation coefficient was used to estimate correlation between episiotomy characteristics and AI, UI and sexual problems for the groups combined. In a subgroup analysis of the women without OASIS, a two-sample t-test was used to compare mean differences in St. Mark’s score between 3a and 3b versus 3c and 4 injuries. A two-sided 5% significance level was used. The study was approved by the Regional Ethics Committee of North Norway (163/2008).

## Results

Demographic data are presented in Table 1. Table 2 shows that anal incontinence was significantly more frequent in women with OASIS compared to women without OASIS, 14 (38%) vs. three (8%), p = 0.05 (OR 4.66, 95% CI 1.34-16.33). In women with OASIS, eight (22%) reported mild AI and six (16%) moderate/severe AI (Table 2). In the group with no OASIS, there were two (5%) women reporting mild AI and one (3%) moderate/severe AI. We observed a significant difference in AI symptoms between women with grade 3c and 4 compared to those with grade 3a and 3b tears. The mean St. Mark’s score was 3.9 higher for women with 3c and 4 injury compared to those with 3a and b injury (95% CI for mean difference, 0.85-7.01, p = 0.01). Although not significant 10 (27%) women with OASIS vs. 3 (8%) women without OASIS p = 0.07 (OR 3.30, 95% CI 0.92-12.1) reported both AI and UI incontinence.

**Table 1:** **Demographic characteristics in women with and without OASIS**

	**Women with OASIS (n = 37)**	**Women without OASIS (n = 37)**	p value**
Mother’s age (years) at delivery*	30 (6.3)	29 (6.4)	0.46
Range	19-41	17-41	
Birthweight (g)*	3764 (662)	3376 (472)	0.01
Range	2441-5245	1922-4180	
Head circumference (cm)*	36 (1.6)	35 (1.8)	0.04
Range	32-40	31-38	
Time from birth to assessment (months)*	34.5 (21.6)	25.9 (13.8)	0.06
Range	1.3 -78.2	7.0 – 57.4	
Instrumental delivery*	19 (51)	19 (51)	N/A

*The values are mean (standard deviation) and range or n (%).

**p values from univariate conditional logistic regression.

**Table 2:** **Anal incontinence, urinary incontinence and sexual problems and OR for OASIS in women with episiotomy**

	OASIS, yes (n = 37)*	OASIS, no (n = 37)*	p-value**	OR**	(95% CI)**
**Anal Incontinence**					
St. Mark’s score < 4	23 (62)	34 (92)		1.00	Reference
St. Mark’s score ≥ 4	14 (38)	3 (8)	0.02	4.66	(1.34-16.33)
No AI, St. Mark’s score < 4	23 (62)	34 (92)		1.00	Reference
St. Mark’s score 4 - 8	8 (22)	2 (5)	0.08	4.00	(0.85-18.80)
St. Mark’s score ≥ 9	6 (16)	1 (3)	0.09	6.00	(0.72-49.80)
**Urinary Incontinence**					
No UI, ICIQ-UI SF^#^ = 0	20 (54)	22 (60)		1.00	Reference
UI, ICIQ-UI SF^#^ ≥1	17 (46)	15 (40)	0.60	1.30	(0.46-3.84)
No UI, ICIQ-UI SF^#^ = 0	20 (54)	22 (60)		1.00	Reference
Mild UI, ICIQ-UI SF^#^ 1 - 8	13 (35)	12 (32)	0.74	1.30	(0.35-4.67)
Moderate/severe UI, ICIQ-UI SF^#^ ≥ 9	4 (11)	3 (8)	0.70	1.30	(0.30-5.96)
**Combined anal and urinary incontinence**					
No	27 (73)	34 (92)		1.00	Reference
Yes	10 (27)	3 (8)	0.07	3.30	(0.92-12.1)
**Sexual problem desire**					
Score 5 (no problem)	4 (12)	16 (43)			Reference
Score 4	16 (48)	12 (32)	0.04	6.94	(1.05-45.91)
Score 3	6 (18)	5 (14)	0.12	3.84	(6.99-21.11)
Score 1-2	7 (21)	4( 11)	0.02	7.62	(1.30- 44.64)
**Sexual problem orgasm**					
Score 5 (no problem)	10 (30)	19 (51)			Reference
Score 4	18 (54)	11 (29)	0.36	2.49	(0.35-17.66)
Score 3	2 (6)	5 (13)	0.90	0.89	(0.14-5.90)
Score 1-2	3 (9)	2 (5)	0.07	3.32	(0.88-12.49)
**Sexual problem with pain**					
Score 5 (no problem)	16 (49)	22 (59)			Reference
Score 4	9 (27)	7 (19)	0.13	4.37	(0.63-30.16)
Score 3	1 (3)	6 (16)	0.29	0.30	(0.03-2.83)
Score 1-2	7 (21)	2 (5)	0.21	2.44	(0.61-9.75)

OASIS, Obstetric anal sphincter injury; AI, anal incontinence; UI, urinary incontinence; OR, odds ratio; CI, confidence interval.

*The values are n (%).

**Univariate conditional logistic regression models. ORs are presented as per unit increase in score point or contrasted to a reference level.

^#^International Consultation on Incontinence Modular Questionnaire Urinary Incontinence Short Form.

There were no significant difference in the rate of UI between women with OASIS and women without OASIS p = 0.60 (OR 1.30 95% CI 0.30-5.96). Seventeen (46%) women with, and 15 (40%) without OASIS experienced involuntary urine leakage once a week or less (Table 2).

Women with OASIS reported significantly more problem with sexual desire compared with women without OASIS, p = 0.02 (OR 7.62, 95% CI 1.30- 44.64) (Table 2).

**Figure 2:**
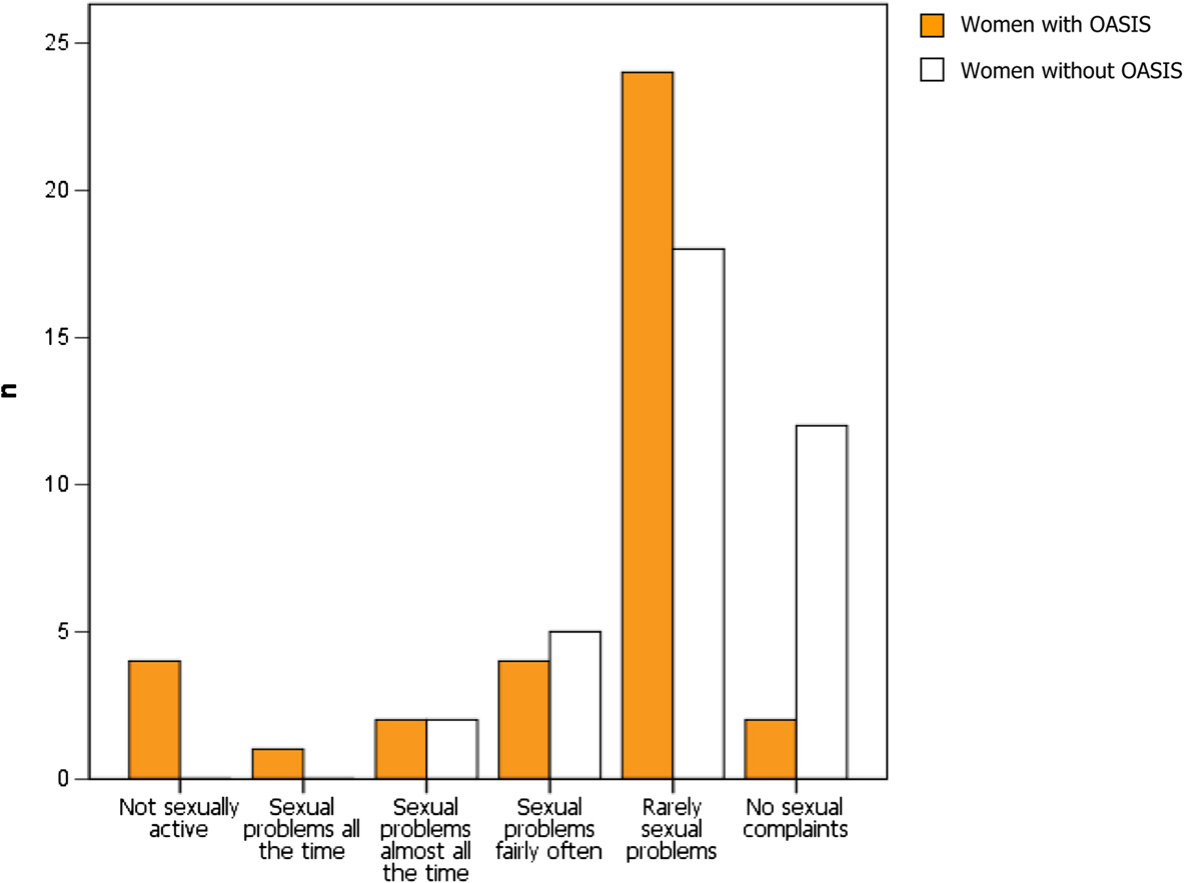
**Combined sexual problems: desire, orgasm and pain (scores) for women with and without OASIS.**

Only two (5.4%) women with OASIS reported no sexual problems compared to 12 (32.4%) in women without. On the other hand, five (13.5%) women with OASIS compared to no (0%) women without OASIS reported no sexual activity or sexual problems all the time (Figure 2).

The episiotomy characteristics varied greatly across the cohort. While we investigated dysfunctions by comparing women with OASIS with women without OASIS, we chose to assess the association between episiotomy characteristics with dysfunctions in all 74 women together. Episiotomy length E ranged from 0.5 cm to 3.2 cm (mean = 1.5, SD = 0.7) and episiotomy depth B ranged from 0.0 cm to 3.0 cm (mean = 1.4, SD = 0.6). Table 3 shows episiotomies categorized according to incision point and width of angle. Sixty-five percent had a medial incision point (hour = 6, and 6–5) and 35% were classified as lateral incision point (hour ≥5).

**Table 3:** **Episiotomy characteristics based on incision point and angle range in 74 primiparous women**

	***Frequency***	***Percent***
Incision point*		
Hour 6	10	14
Hour 6-5	38	51
Hour 5	15	21
Hour 5-4	9	12
Hour 4	1	1
Hour 3	1	1
Angle range		
0-15	7	10
16-30	22	30
31-45	18	24
46-60	15	20
61+	12	16

*according to an analog clock display.

Table 4 shows Spearman’s correlation coefficients (r) between dysfunction score and episiotomy characteristics. Two out of 12 coefficients were significant: a moderate degree of correlation was observed for total sexual problem score with episiotomy length and depth (r = 0.28 and 0.31, respectively).

**Table 4:** Correlation between dysfunction score and episiotomy characteristics in 74 primiparous women*

	**Episiotomy characteristics**			
**Dysfunctions**	**Episiotomy length - E**	**Episiotomy depth – B**	**Episiotomy insertion point – D**	**Episiotomy angle**
AI (St.Mark’s-score)	−0.14	−0.03	-0.07	0.05
UI (ICIQ UI SF)	−0.04	0.06	0.03	0.01
Sexual problem (total score)	0.28**	0.31**	0.08	0.04

*Spearman’s Rho coefficient is used to assess correlations.

**p < 0.05.

## Discussion

Women with OASIS reported significantly more AI and problem with sexual desire compared to women without OASIS. Episiotomy characteristics varied extensively between the women. We found no association between episiotomy characteristics and AI, UI. Episiotomy length (E) and depth (B) were associated with sexual problems. The correlation is positive, meaning that the episiotomies with protective characteristics of length and depth were associated with fewer problems.

### Obstetric anal sphincter injuries in relation to AI and sexual problems

OASIS have consistently been associated with increased rate of AI and sexual problems. The mean rate of AI has been reported to be 39% (range 15-61%) after primary repair [1,4,28] although a recent report suggest that AI following OASIS can be minimized with appropriate repair by trained doctors [28]. Sexual problems after vaginal delivery in women sustaining OASIS have been reported to be 29-39% [29,30] compared to13-19% for women with no OASIS [29,30]. We found that problems with desire were significantly more prevalent for women with OASIS, whereas problems with orgasm and pain were not significantly different between the groups.

The association we found between degree of injury and severity of AI symptoms further highlights that the degree of perineal damage is one of the key factors of postpartum pelvic floor dysfunction and is supported by earlier studies. Roos et al. [3] reported significantly poorer outcomes for women sustaining OASIS grades 3c and 4 than grades 3a and 3b, and de Leeuw et al. [1] found that the odds of developing AI significantly increased according to increased severity of injury.

### Episiotomy characteristics in relation to AI and sexual problems

We found that all the episiotomy characteristics varied across the cohort. This corroborates with previous studies [9,11,12]. We have previously reported that specific characteristics were associated with decreased risk of sustaining OASIS [18]. This study found that there were no association between the episiotomy characteristic that decreased the risk of OASIS and increased dysfunctions such as AI, UI and sexual problems.

To the best of our knowledge only a few studies have looked at the actual episiotomy technique used in relation to pelvic floor dysfunctions. Episiotomy technique during delivery was assured to have an angle of 60° in 60 primiparous women in a prospective study [31]. There was no OASIS in this group. After six months 51 women registered their symptoms of anal incontinence and perineal pain. Two women (4%) registered symptoms of AI, whereas episiotomy related perineal pain was reported by seven (14%) women. Fodstad et al. [16] assessed the episiotomy 0–3 days after birth in 300 women and investigated perineal pain and blood loss. The episiotomies were classified as midline, mediolateral, and lateral and no difference in perineal pain and blood loss in relation to technique were found.

### High rates of UI in both groups

This study reports high rates of UI in both groups and no significant difference which corroborated with previous results of Borello-France et al. [7] and de Leeuw et al. [1], indicating that the development of UI after delivery is caused by different anatomical injuries than OASIS.

### Study limitations

The current study has limitations. It is a small retrospective study, and the results must therefore be interpreted with caution. Independent predictors such as pre-pregnancy AI, UI, and sexual complaints are unknown [32]. Follow-up time is another limitation since all described functions vary with time. Even though the mean follow-up time was not significantly different between the groups, the range was larger in women with OASIS compared to women without. Our study did not have a control group with an intact perineum, simply because it was too difficult to match the criteria of 51% instrumental delivery without episiotomy. Finally, birthweight was significantly higher in the case group compared to the control group and might be a potential confounder. Although birthweight has been reported to be a risk factor for OASIS [33], birthweight is not independently associated with AI [34,35]. As far as we know, birthweight has not been independently associated with either sexual problems or UI.

## Conclusion

OASIS is the primary risk factor for AI and sexual problems in primiparous women with episiotomy. We did not find any association between episiotomy with protective characteristics and postpartum AI, UI and increased sexual problems. A correctly performed episiotomy may prevent OASIS. This study highlights that the sequelae after episiotomy with preventive characteristics is not as bad as having a sphincter injury.

## Abbreviations

OASIS: Obstetric anal sphincter injuries
UI: Urinary incontinence
AI: Anal incontinence
OR: Odds ratio
